# Induction of Ankrd1 in Dilated Cardiomyopathy Correlates with the Heart Failure Progression

**DOI:** 10.1155/2015/273936

**Published:** 2015-04-16

**Authors:** Julius Bogomolovas, Kathrin Brohm, Jelena Čelutkienė, Giedrė Balčiūnaitė, Daiva Bironaitė, Virginija Bukelskienė, Dainius Daunoravičus, Christian C. Witt, Jens Fielitz, Virginija Grabauskienė, Siegfried Labeit

**Affiliations:** ^1^Department of Integrative Pathophysiology, Medical Faculty Mannheim, Theodor-Kutzer-Ufer 1-3, 68167 Mannheim, Germany; ^2^Department of Pathology, Forensic Medicine and Pharmacology, Faculty of Medicine, Vilnius University, M. K. Ciurlionio g. 21, LT-03101 Vilnius, Lithuania; ^3^Vilnius University Hospital Santariskiu Klinikos, Santariškiu g. 2, LT-08661 Vilnius, Lithuania; ^4^State Research Institute, Center for Innovative Medicine, Department of Stem Cell Biology, Žygimantu g. 9, LT-01102 Vilnius, Lithuania; ^5^Vilnius University Institute of Biochemistry, Mokslininku g. 12, LT-08660 Vilnius, Lithuania; ^6^Experimental and Clinical Research Center (ECRC), Max-Delbrueck Center for Molecular Medicine (MDC), Robert-Rössle-Straße 10, Buch, 13125 Berlin, Germany

## Abstract

Progression of idiopathic dilated cardiomyopathy (IDCM) is marked with extensive left ventricular remodeling whose clinical manifestations and molecular basis are poorly understood. We aimed to evaluate the clinical potential of titin ligands in monitoring progression of cardiac remodeling associated with end-stage IDCM. Expression patterns of 8 mechanoptotic machinery-associated titin ligands (*ANKRD1*, *ANKRD2*, *TRIM63*, *TRIM55*, *NBR1*, *MLP*, *FHL2*, and *TCAP*) were quantitated in endomyocardial biopsies from 25 patients with advanced IDCM. When comparing NYHA disease stages, elevated *ANKRD1* expression levels marked transition from NYHA < IV to NYHA IV. *ANKRD1* expression levels closely correlated with systolic strain depression and short E wave deceleration time, as determined by echocardiography. On molecular level, myocardial *ANKRD1* and serum adiponectin correlated with low *BAX/BCL-2* ratios, indicative of antiapoptotic tissue propensity observed during the worsening of heart failure. ANKRD1 is a potential marker for cardiac remodeling and disease progression in IDCM. *ANKRD1* expression correlated with reduced cardiac contractility and compliance. The association of *ANKRD1* with antiapoptotic response suggests its role as myocyte survival factor during late stage heart disease, warranting further studies on ANKRD1 during end-stage heart failure.

## 1. Introduction

Despite intensive search for therapeutic interventions, idiopathic dilated cardiomyopathy (IDCM) remains the major cause of heart failure eventually leading to heart transplantation. Limited availability of donor hearts results in long waiting times before transplantation can be performed. Many patients with end-stage heart failure perish before a donor heart becomes available. Management of patients awaiting transplantation is demanding, because some of them remain stable while others deteriorate quickly [1]. However, transplantation specialists do not have reliable tools to differentiate between these two disease courses. Therefore, there is a pressing need for markers predicting the prognosis and disease course of end-stage heart failure caused by IDCM in order to prioritize patient listing for transplantation.

Myocyte apoptosis was shown to be a contributor to the development of heart failure (HF) [2], whereas experimental studies on mouse models have suggested that this process might at least be mediated trough the titin filament and its ligands [3]. However, this hypothesis has not been tested so far in clinical settings. Here, we have evaluated expression levels of 8 titin ligands in endomyocardial biopsies (EMB). Differences in* ANKRD1* expression pattern were found to be most informative:* ANKRD1* levels were associated with decreased cardiac contractility and compliance.* ANKRD1* gene encodes an ankyrin repeat-domain containing protein 1 (Ankrd1, known as well as CARP-cardiac ankyrin repeat protein). Ankrd1 belongs to a family muscle ankyrin repeat proteins, interacting with titin in a stretch-dependent manner: upon mechanical stretch it translocates to the nucleus, where it acts as transcription cofactor [4]. Ventricular* ANKRD1* upregulation was observed in increased stretch states such as experimental pressure overload [5] or clinical heart failure due to dilated [6] and arrhythmogenic right ventricular cardiomyopathy [7]. Moreover, mutations in* ANKRD1* were found to be associated with dilated [8] and hypertrophic cardiomyopathy [9]. Functionally, myocardial ANKRD1 acts as an antiapoptotic survival factor after ischemia-reperfusion injury [10] and hypoxia [11] and is downregulated in apoptosis-driven [12, 13] anthracycline cardiomyopathy [14]. In this work, we present data indicating that ANKRD1 together with adiponectin might act as myocyte survival factors, associated with antiapoptotic response in the terminal stage of IDCM.

## 2. Materials and Methods

### 2.1. Patients

Our study cohort was composed of patients admitted to the Vilnius University Hospital during 2011–2013 with suspected diagnosis of IDCM. All patients underwent a careful history and physical examination, as well as routine laboratory studies, including B-type natriuretic peptide (BNP), adiponectin, and cardiac troponin T (hsTnT). 23 patients were selected because of a reduced left ventricular ejection fraction (LVEF < 45%) in the absence of significant coronary artery disease (stenosis of coronary arteries of less than 50%), a history of myocardial infarction, and other specific heart muscle diseases (primary valvular heart disease, toxic cardiomyopathy, arterial hypertension, renal failure, and abuse of alcohol or illicit drugs), all consistent with primary IDCM. IDCM diagnosis was confirmed by histological analysis of endomyocardial biopsies (EMB). Patients who were diagnosed as having acute myocarditis according to histological evidence were excluded from the present study. NYHA class was assigned by a clinician unaware of patient echocardiographic investigation. All patients received maximal pharmacological heart failure therapy according to European Society of Cardiology guidelines: ACE inhibitors or angiotensin receptors blockers, *β*-blockers, mineralocorticoid receptors blockers, digitalis (in case of atrial fibrillation), diuretics, anticoagulant (in case of atrial fibrillation, EF < 40%), and antiarrhythmics (class III: amiodarone) (see Supplementary Table  1 in Supplementary Material available online at http://dx.doi.org/10.1155/2015/273936). Clinical decision about possible treatment with cardiac resynchronization therapy, radiofrequency ablation, or implantation of a left ventricular assist device or implantable cardioverter-defibrillator was made after coronary angiography and EMB. All patients gave written informed consent to this study, including cardiac catheterisation and EMB. The study was approved by Lithuanian Bioethics Committee (Protocol number 158200-2011/09) and conducted in compliance with the Declaration of Helsinki.

### 2.2. Echocardiography and Cardiac Catheterisation

Echocardiographic evaluation was performed 1 day before cardiac catheterisation by GE Vivid 7 and 9 ultrasound system by an investigator blinded for the study objectives. The standard LV apical (apical 4, apical 2, and apical 3) views and parasternal short axis views at mid-papillary level were acquired at 70–90 frames/s. Conventional echocardiographic parameters such as left ventricular ejection fraction (LVEF), left ventricular end-diastolic dimension (LVEDD), left ventricular end-systolic dimension (LVESD), velocities of E and A waves (E and A) and their ratio (E/A), and E deceleration time (DcT) were obtained. All images were stored digitally for subsequent offline analysis. Quantification of myocardial deformation values was performed by 2D speckle tracking using Echopac PCBT08 (GE Healthcare) software. After the manual selection, speckles were assumed automatically and then confirmed by the investigator. By the semiautomatic postprocessing longitudinal (in 4 chamber-4C, two-chamber-2C, and three chamber-3C views), circumferential, and radial strain (RS) and strain rate parameters were extracted. Mean pulmonary artery (PA) pressure, pulmonary capillary wedge pressure (PCWP), and pulmonary vascular resistance (PVR) were measured and EMB was taken during right heart catheterization. Biopsy specimens were immediately placed to −70°C until further processing.

### 2.3. Quantitative RT-PCR

RNA from EMB samples was extracted using RNeasy fibrous tissue minikit according to provided protocol (Qiagen). Tissue was directly homogenized in lysis buffer using Ultra-Turrax device. RNA was reverse transcribed using High Capacity RNA-to-cDNA Kit primed with mixture of random and poly-dT primers (Invitrogen). Transcripts were quantified using TaqMan Gene Expression assay on Real-Time Stratagene MX 3005P machine following manufacturer recommendations. Amplification efficiency validated TaqMan probes (Supplementary Table  2) used in this work are presented in Table 2. 18S rRNA was used for standardization. As this study did not contain a reference group and was based on individual EMB samples, *C*_*t*_ method could not be used for quantification of transcript levels. Therefore relative transcript abundances were quantified using *C*_*t*_ method (*C*_*t*  gene  of  interest_ − *C*_*t*  S18  rRNA_). Transcript levels in this work are expressed as negative *C*_*t*_ values; thus higher −*C*_*t*_ values denote higher mRNA levels whereas negative *C*_*t*_ values represent genes that are less abundant compared with the reference gene. Bax/Bcl-2 ratios were calculated as *C*_*t*_((*C*_*t*  Bax_ − *C*_*t*  S18  rRNA_)−(*C*_*t*  Bcl-2_ − *C*_*t*  S18  rRNA_)) corresponding to relative expression ratio [15].

### 2.4. Measurement of Activated Caspase-3

Levels of activated caspase-3 in EMB samples were determined using ELISA, specific for the activated protein form (Invitrogen, Paisley, UK). Tissue samples were lysed by sonification in RIPA lysis buffer (Thermo Scientific Inc., USA) supplemented with phosphatase and protease inhibitors according to manufacturer's recommendations (Thermo Scientific Inc., USA). Protein content in clarified lysates was measured using modified Lowry protein assay using bovine serum albumin as standard according to the provided protocol (Thermo Scientific Inc., USA). Analyte concentration was expressed as ng/mg of total protein.

### 2.5. Statistics

Statistical analysis was performed using SPSS 17 software. Nonparametric Mann-Whitney *U* test was used to assess differences between two independent groups. Pearson product-moment correlation coefficient was used to evaluate linear dependence between values. If otherwise not indicated, a value of *P* < 0.05 was taken as significant (two*-*tailed).

## 3. Results

### 3.1. Induction of the Titin Ligands* ANKRD1*,* ANKRD2,* and* TRIM63* in Patients with End-Stage IDCM in Correlation with NYHA Staging

**Figure 1:**
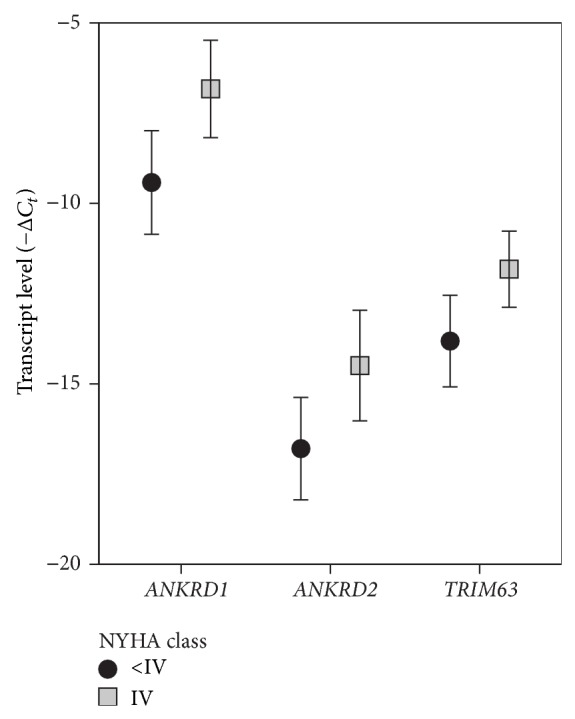
Myocardial titin ligand expression patterns associate with disease progression in IDCM patients. Statistically significant titin ligand expression differences represented as bar graph. Data are presented as mean ± 2 s.e.m. Note the highest expression level and the most pronounced difference between groups in* ANKRD1* expression pattern.

Here, we determined the transcript levels of* ANKRD1, ANKRD2, TRIM63, TRIM55, NBR1, MLP, FHL2,* and* TCAP* in EMB biopsies from end-stage IDCM patient cohort to test their potential roles in titin-based cellular stress transmission. Clinically, we surveyed patients with significantly reduced LVEF, elevated BNP and TnT values, elevated intracardiac pressures, and impaired relaxation (Table 1). Patients were divided into two groups according to the severity of HF symptoms based on NYHA functional class: Group IV (symptomatically Severe HF) and Group <IV (mainly class III; symptomatically moderate HF), and the expression levels of mechanoptotic machinery members were compared (Table 2). Out of 8 studied transcripts,* ANKRD1, ANKRD2,* and* TRIM63* were significantly higher in NYHA class IV than <IV NYHA class group (Figure 1, *P* = 0.01 for* ANKRD1* and *P* = 0.03 for* ANKRD2* and* TRIM63*).* ANKRD1* had the highest expression levels compared to other titin ligands and the most profound 6-fold induction (calculated as 2^*C*_*t*_  IV  NYHA−*C*_*t*_<IV  NYHA^) in NYHA IV patients as compared to NYHA <IV patients. Therefore,* ANKRD1* expression pattern was the most sensitive to the disease progression and was chosen for the further analysis.

### 3.2. *ANKRD1* Expression Correlates with LV Remodeling in End-Stage IDCM Patients

**Figure 2:**
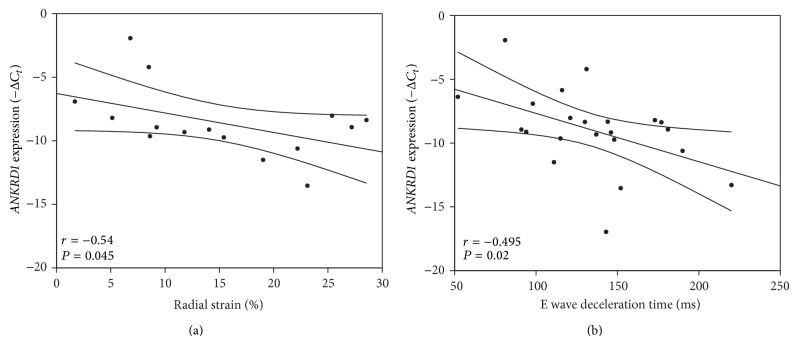
Increased* ANKRD1* expression marks left ventricular remodeling. (a) Linear correlation between* ANKRD1* expression and radial stain. Regression line is represented within 95% confidence interval for the mean value. A negative correlation between* ANKRD1* expression (−*C*_*t*_) and radial strain (RS; %) was found (*n* = 14, *r* = −0.54, *P* < 0.05). (b) Linear correlation between* ANKRD1* expression and E wave deceleration time. Regression line is represented within 95% confidence interval for the mean value. A negative correlation between* ANKRD1* expression (−*C*_*t*_) and E wave deceleration time (ms) was detected (*n* = 21, *r* = −0.495, *P* < 0.05).

In order to investigate clinical correlates of* ANKRD1* expression we looked for further clinical parameters different between NYHA class >IV and <IV. Statistically significant differences between NYHA <IV and NYHA IV groups were only detected for parameters of systolic strain (Table 3). Strain measurements quantify magnitudes and velocities of myocardial deformation estimating myocardial contractility [16]. Radial and longitudinal strain in 3C projection showed marked reduction in severe HF patients (NYHA IV) when compared to symptomatically moderate HF patients (NYHA <IV) (Table 3). Correlation analysis confirmed that reduced cardiac contractility correlated with* ANKRD1* expression independently from NYHA functional class (Figure 2(a)). Further, we found that* ANKRD1* expression correlated with the E wave deceleration time shortening, which is an index for LV stiffness [17] (Figure 2(b)). Taken together,* ANKRD1* expression is associated with LV remodeling resulting in reduced cardiac contractility and compliance.

### 3.3. Deteriorating Cardiac Contractility Is Associated with Blunted Myocardial Vulnerability to Apoptosis

**Figure 3:**
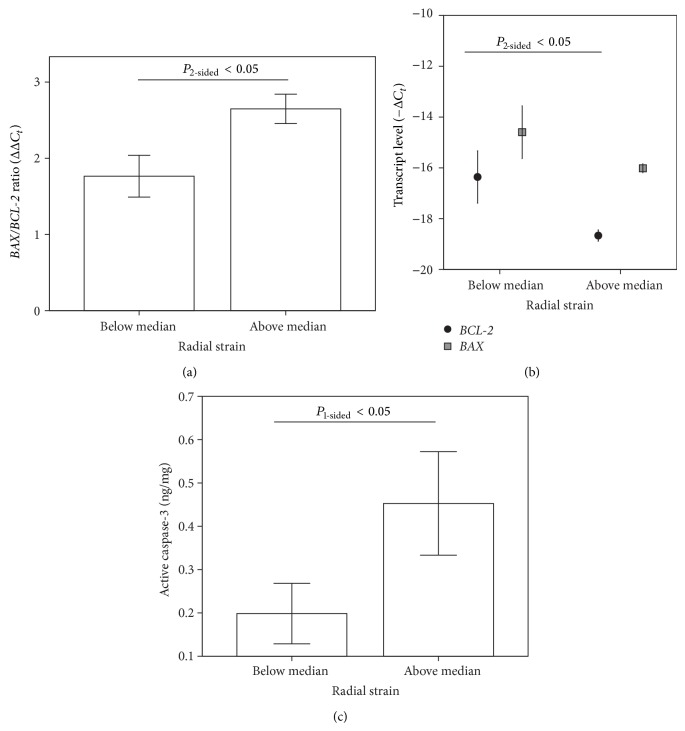
*ANKRD1* and adiponectin upregulation are associated with antiapoptotic response in progression of IDCM. (a) Difference in* BAX/BCL-2* ratio between high and low RS groups. Data are presented as mean ± 2 s.e.m; *P* value < 0.05 (Mann*-*Whitney *U* test). (b) Relative expression levels of* BAX* and* BCL-2*. Data are presented as mean ± 2 s.e.m. *P*_one-sided_ value < 0.05 (Mann*-*Whitney *U* test). (c) Difference in active caspase-3 levels between high and low RS groups. Data are presented as mean ± 2 s.e.m; *P*_one-sided_ value < 0.05 (Mann*-*Whitney *U* test); *n* = 6 (lower RS); *n* = 5 (higher RS).

**Figure 4:**
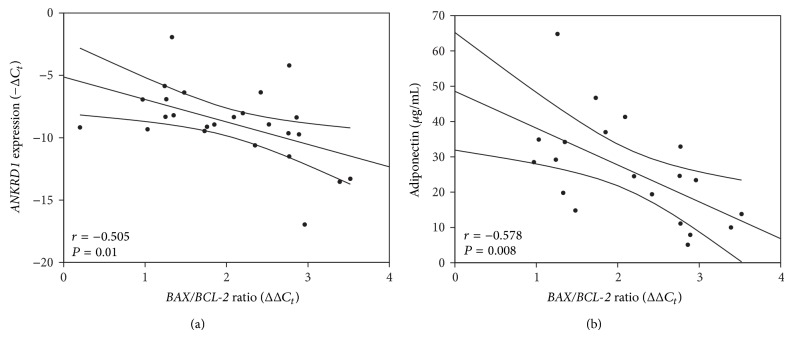
*ANKRD1* and adiponectin correlate with tissue propensity to apoptosis. (a) Linear correlation between* ANKRD1* expression and* BAX/BCL-2* ratio. Regression line is represented within 95% confidence interval for the mean value. A negative correlation between* ANKRD1* expression (−*C*_*t*_) and* BAX/BCL-2* ratio (*C*_*t*_) was found (*n* = 25, *r* = −0.505, *P* = 0.01). (b) Linear correlation between adiponectin levels and* BAX/BCL-2* ratio. Regression line is represented within 95% confidence interval for the mean value. A negative correlation between serum adiponectin levels (*μ*g/mL) and cardiac* BAX/BCL-2* ratio (*C*_*t*_) was observed (*n* = 20, *r* = −0.578, *P* < 0.01).

Because myocyte apoptosis is associated with contractile dysfunction in HF [18, 19] and ANKRD1 acts as antiapoptotic [10, 11], we hypothesized that decreased cardiac contractility in end-stage IDCM patients is associated with proteins involved in mechanoptosis. As presented above, the most pronounced deterioration of cardiac contractility upon IDCM progression was observed in radial direction by echocardiography, referred to as radial strain (RS). Thus, for further analysis we used RS to monitor worsening of cardiac contractility. Patients were subdivided into two groups according to the median value of RS. The above median RS group displayed normal radial cardiac contractility as mean RS was still within a healthy population reference range [20]. In contrast, patients with RS values below the median RS had severely impaired cardiac contractility (Table 4). Further strain parameters, RS rate and 3C longitudinal strain, indicated better cardiac contractility in above median RS group. None of the echocardiographic parameters or cardiac chamber pressure values reached statistically significant differences. However, the group with severe loss of radial deformation (below RS median) showed a tendency towards worse cardiac function.* ANKRD1* and stretch-marker BNP levels were higher in the group with impaired radial contractility. Next, we evaluated transcript levels of proapoptotic* BAX* and antiapoptotic* BCL-2* whose ratio defines tissue propensity to apoptosis [21] and amount of active caspase-3, a major apoptosis executer [22] that corresponds to intensity of ongoing apoptosis. Better contractility correlated with lower antiapoptotic* BCL-2* levels and thus corresponded to a group more prone to apoptosis (Figures 3(a) and 3(b)). Consequently, higher levels of ongoing apoptosis, as measured by levels of active caspase-3, were detected in higher RS group (Figure 3(c)). Low* BAX/BCL-2* ratios and therefore myocardial insensitivity to apoptosis correlated well with* ANKRD1* transcript levels (Figure 4(a)). In addition,* BAX/BCL-2* ratios inversely correlated with serum adiponectin levels (Figure 4(b)). Taken together our data imply that* ANKRD1* might be involved in antiapoptotic response observed in end-stage DCM [23].

## 4. Discussion

Genes coding for titin binding proteins has been suggested to act as members of a titin filament based stress sensing mechanoptotic machinery in previous mouse work [3]. Here, we tested for a potential clinical significance of titin ligands for LV remodeling in IDCM patients. Out of 8 studied transcripts we found that* ANKRD1* expression levels showed the most significant increase in symptomatically severe HF (NYHA class IV) compared to moderate HF (NYHA < IV) patients. Clinically, severe HF patients had notably poorer systolic strain rates indicating reduced cardiac contractility. Our data indicate that myocardial strain parameters are superior to LV ejection fraction and chamber diameter, intracardiac pressure, and relaxation measures in detecting the severity of heart failure as estimated by NYHA functional class. Our findings are in line with previous studies where myocardial strain predicted rapid HF progression in end-stage IDCM patients [1]. We found a significant reduction of longitudinal strain in 4C and 3C projections, but the major difference was observed for radial strain measurements. These findings are in line with a study on hypertensive patients with heart failure, where a reduction in radial strain was only seen in NYHA classes III-IV, whereas longitudinal strain was decreased as early as NYHA class II [24]. Moreover we found that* ANKRD1* expression correlated not only with reduced LV contractility, but also with increased cardiac stiffness;* ANKRD1* expression positively correlated with shortening of E wave deceleration time, marking restrictive filling pattern—the most powerful independent prognostic indicator of poor outcome or transplantation in DCM patients [25]. Taken together our data indicate that ventricular* ANKRD1* levels in IDCM patients are associated with progression of LV remodeling, resulting in reduced cardiac contractility and compliance. In DCM, myocyte apoptosis is related to LV dysfunction [26] and appears to directly affect cardiac contractility [18, 19]. Finally, myocardial ANKRD1 functions as an antiapoptotic survival factor after ischemia-reperfusion injury [10] and hypoxia [11]. Therefore, we investigated the relation between* ANKRD1* expression and apoptotic status in the myocardium. We found that tissue samples more susceptible to apoptosis (high* BAX*/*BCL-2* ratio) had lower* ANKRD1* levels than apoptosis-resistant samples (low* BAX*/*BCL-2* ratio) implying that* ANKRD1* could act as myocyte survival factor. However, counterintuitively the group with less impaired cardiac contractility (above median RS) was more prone to apoptosis than low RS group. In addition, impaired contractility was associated with higher* BAX*/*BCL-2* ratios and elevated levels of key apoptosis-executing enzyme [22] and active caspase-3, indicating that better contractility was marked with higher levels of ongoing apoptosis. These findings correspond to previous observations that terminal IDCM stage is associated with marked antiapoptotic response [23]. In agreement with previous data on end-stage HF [27], we found that a decreased* BAX*/*BCL-2* ratio in hearts with severely impaired contractility was mainly due to increased levels of the survival factor Bcl-2. Moreover, insensitivity to apoptosis was associated with increased serum adiponectin levels, a predictor for mortality in patients with chronic HF [28]. Possibly, adiponectin could account for the reduced* BAX*/*BCL-2* ratio in end-stage IDCM patients, as it has antiapoptotic effects in myocardium [29].

**Figure 5:**
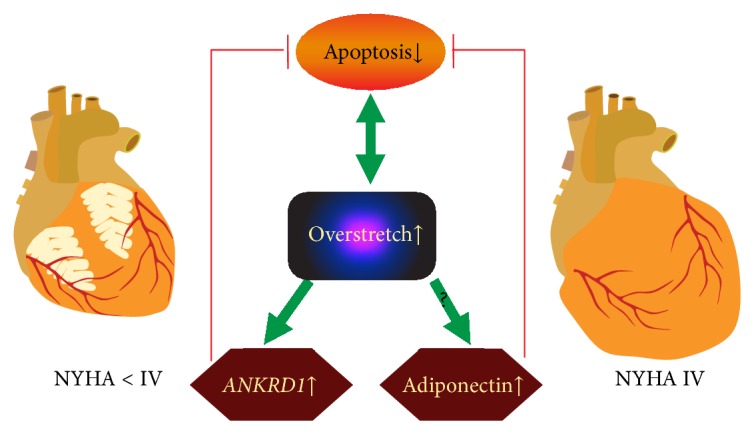
Antiapoptotic response in end-stage IDCM. Speculatively, ongoing apoptosis and myocyte overstretch in vicious cycle mediate the LV remodeling, whereas myocyte stretch induces Ankrd1 expression and increases adiponectin levels by unknown mechanism that in turn inhibit apoptotic signaling. Arrows indicate process dynamics in NYHA <IV to NYHA IV transition.

Speculatively, stretch-sensing and prosurvival properties of Ankrd1 could be responsible for the observed antiapoptotic response in terminal IDCM stages (Figure 5). LV remodeling in IDCM leading to the wall thickening and chamber dilation is accompanied by myocyte overstretch and slippage [30] which in vicious cycle provokes myocyte mechanoptosis [31]. Subsequently stretch would directly upregulate* ANKRD1* transcript and launch Ankrd1-mediated survival cascades. Hypothetically,* ANKRD1* and adiponectin or their agonists could be used as heart-specific antiapoptotic agents in treatment of IDCM.

This study has some limitations which have to be pointed out. The study cohort consisted of patients with advanced HF (NYHA classes III-IV); thus future research would be needed to confirm the validity of observed clinical correlations in patients with mild HF (NYHA I-II). Recent studies have demonstrated that genetic alterations of titin [32] and* ANKRD1* [33, 34] are associated with DCM and result in poorer prognosis [32]. Thus, it is not excluded that genetic alterations of titin-ligand network might be present in studied patients. However, observed upregulation of* ANKRD1* is very likely to be universal pathophysiological as it was described in controlled increased stretch states such as experimental pressure overload [5] and not due to genetic alterations. This study was based on patients with advanced stages of HF (mostly NYHA classes III-IV); thus future research would be needed to evaluate the validity of observed clinical correlations in early HF (NYHA I-II).

Although transcript levels do not always represent changes in protein concentration, our data suggest that* ANKRD1* transcript quantification is sensitive test for monitoring progression of advanced IDCM stages. Moreover, quantitative RT-PCR might be method of choice to quantify* ANKRD1* levels, when using minute EMB samples.

In conclusion, expression profiling of selected mechanoptotic machinery members revealed association between increased* ANKRD1* expression and deterioration of cardiac contractility and compliance in IDCM patients. Therefore, elevated* ANKRD1* expression could serve as potential clinical marker to uncover a coming need to plan heart transplantation in end-stage HF patients. Further research is warranted on the functional roles of* ANKRD1* induction in IDCM associated apoptosis.

## Supplementary Material

Supplementary Table 1 Pharmacological heart failure therpay of studied cohort. Supplementary Table 2 List of Taqman probes used in this study.

## Figures and Tables

**Table 1:** Patient characteristics.

	NYHA class	
	<IV	IV
Age (years)	44.2 ± 14.2	43.5 ± 13.9
Sex		
Female	4	1
Male	15	5
BMI (kg/m^2^)	24.7 ± 4.7	28.7 ± 5.1
NYHA class		
II	1	
III	16	
III-IV	2	
IV		6
BNP (pg/mL)	1259 ± 1180	1964 ± 1177
TnT (pg/mL)	80.9 ± 143.5	34.4 ± 13.9
Adiponectin (*μ*g/mL)	22.9 ± 12.6	33.8 ± 17.5
LVEF (%)	24.1 ± 7.4	20.1 ± 6.2
LVESD (mm)	58.2 ± 9.9	64.3 ± 10.2
LVEDD (mm)	67.3 ± 7.4	71.1 ± 12
RAP (mmHg)	12.5 ± 7.3	16.2 ± 11
PAP (mmHg)	32.2 ± 12.1	43.2 ± 15.8
PCWP (mmHg)	23 ± 9.1	31.8 ± 14.2
PVR (Wood units)	2.5 ± 1.7	3.2 ± 0.8
E wave (m/s)	0.9 ± 0.2	1 ± 0.3
A wave (m/s)	0.5 ± 0.2	0.4 ± 0.1
E/A	1.8 ± 1.1	2.3 ± 0.4
E wave *T*_dec_ (ms)	140.1 ± 35.2	109 ± 45.4

**Table 2:** Differences in myocardial expression patterns of titin ligands according to NYHA functional class <IV (*n* = 19) and NYHA IV (*n* = 6).

Transcript level (−Δ*C*_*t*_)	NYHA class		*P* value
	<IV	IV	
*ANKRD*1^∗^	−9.42 ± 0.7	−6.83 ± 0.7	0.01
*ANKRD*2^∗^	−16.79 ± 0.7	−14.5 ± 0.8	0.03
*TRIM*63^∗^	−13.81 ± 0.6	−11.82 ± 0.5	0.03
*TRIM*55	−14.23 ± 0.8	−13.11 ± 0.7	0.25
*NBR*1	−11.97 ± 0.7	−11.73 ± 0.9	0.44
*MLP*	−10.39 ± 1.0	−9.15 ± 0.9	0.20
*FHL*2	−10.07 ± 0.8	−9.75 ± 0.6	0.56
*TCAP*	−7.08 ± 0.9	−6.16 ± 0.8	0.30
*BCL-*2^∗^	−17.81 ± 0.4	−16.25 ± 0.7	0.02
*BAX*	−15.71 ± 0.3	−14.39 ± 0.9	0.22

Significant differences (*P* value < 0.05 of the Mann*-*Whitney *U* test) marked with ∗.

**Table 3:** Differences in strain parameters between NYHA <IV and NYHA IV groups.

Strain parameter	NYHA class		*P* value
	<IV	IV	
4C strain (%)	−7.4 ± 1	−4 ± 0.8	0.10
4C strain rate (s^−1^)	−0.4 ± 0	−0.2 ± 0	0.10
2C strain (%)	−6.7 ± 0.9	−5.1 ± 0.9	0.25
2C strain rate (s^−1^)	−0.3 ± 0	−0.3 ± 0	0.34
3C strain (%)^∗^	−8.1 ± 0.8	−3.9 ± 1.1	0.01
3C strain rate (s^−1^)	−0.8 ± 0.4	−0.2 ± 0	0.11
Circumferential strain (%)	−5.5 ± 0.5	−4.3 ± 1.2	0.51
Circumferential strain rate (s^−1^)	−0.4 ± 0	−0.2 ± 0	0.20
Radial strain (%)^∗^	17.4 ± 2.3	6.1 ± 1.7	0.01
Radial strain rate (s^−1^)	1.3 ± 0.1	0.8 ± 0.2	0.11

Note further reduction of systolic strain parameters along disease progression.

Significant differences (*P* value < 0.05 of the Mann-Whitney *U* test) marked with ∗.

**Table 4:** Clinical characteristics of patients (*n* = 14) with high radial strain (RS, above median) and low RS (below median).

	Radial strain	
	Below median	Above median
Age (years)	48 ± 6.51	44 ± 3.3
Sex		
Female	2	3
Male	5	4
BMI (kg/m^2^)	25.6 ± 2.0	27.03 ± 2.13
NYHA class		
III	3	5
III-IV	0	2
IV	4	0
BNP (pg/mL)^∗^	1824 ± 461	525 ± 197
TnT (pg/mL)	31.17 ± 6.1	157 ± 136.4
Adiponectin (*μ*g/mL)^∗^	35 ± 5.4	8.5 ± 1.8
LVEF (%)	21.29 ± 1.1	25.29 ± 3.34
LVESD (mm)	61 ± 2.1	55.5 ± 2.1
LVEDD (mm)	71.3 ± 2.1	65.6 ± 1.7
RAP (mmHg)	12.33 ± 3.9	9.33 ± 1.28
PAP (mmHg)	32.6 ± 4.7	25.3 ± 1.91
PCWP (mmHg)	23.14 ± 3.8	18.17 ± 1.51
PVR (Wood units)	2.5 ± 0.44	2.025 ± 0.55
E wave (m/s)	1 ± 0.11	0.92 ± 0.06
A wave (m/s)	0.398 ± 0.05	0.63 ± 0.095
E/A	2.71 ± 0.36	1.86 ± 0.48
E wave *T*_dec_ (ms)	118 ± 12	146 ± 15.42
3C strain (%)^∗^	−5.07 ± 0.96	−8.75 ± 0.98
Radial strain (%)^∗^	7.39 ± 1.23	21.1 ± 1.95
Radial strain rate (s^−1^)^∗^	0.88 ± 0.19	1.55 ± 0.13
*ANKRD1* (−Δ*C*_*t*_)^∗^	−7.02 ± 1.1	−10.26 ± 0.68
*BCL-2* (−Δ*C*_*t*_)^†^	−16.36 ± 1.0	−18.67 ± 0.22
*BAX* (−Δ*C*_*t*_)	−14.60 ± 1.0	−16.02 ± 0.17
*BAX*/*BCL-2* ratio (ΔΔ*C*_*t*_)^∗^	1.76 ± 0.27	2.65 ± 0.19
Active caspase-3 (ng/mg)^†^	0.19 ± 0.06	0.45 ± 0.11

^∗^ *P*_two-sided_< 0.05; ^†^ *P*_one-sided_ < 0.05 (Mann*-*Whitney *U* test).
