# Supplementary material for: Induction of Ankrd1 in Dilated Cardiomyopathy Correlates with the Heart Failure Progression
Source: Biomed Res Int. 2015 Apr 16;2015:273936. doi: 10.1155/2015/273936 (PMC4415747; doi:10.1155/2015/273936)
Supplement: Supplementary file 1 — Supplementary Table 1 Pharmacological heart failure therpay of studied cohort. Supplementary Table 2 List of Taqman probes used in this study. [file 273936.f1.docx]

**Induction of Ankrd1 in dilated cardiomyophaty correlates with the heart failure progression**

*Julius Bogomolovas, Kathrin Brohm, Jelena Čelutkienė, Giedrė Balčiūnaitė, Daiva Bironaitė, Dainius Daunoravičus Christian C. Witt, Jens Fielitz, Siegfried Labeit, Virginija Grabauskienė*

**Supplementary tables**

Supplementary table 1 Drug therapy of patients in study cohort

| **% of patients** |
| --- |
| Conventional treatment of heart failure   - - ACE inhibitors 40% (10/25)   - β-blockers 88% (22/25)   - Mineralocorticoid receptor blockers 100% (25/25)   - Digitalis (in atrial fibrillation) 24% (6/25)   - Diuretics 100% (25/25)   - Anticoagulation (atrial fibrillation, EF < 40%) 68% (17/25)   - Antiarrhythmics (class III: amiodarone) 32% (8/25) |

Supplementary table 2 TaqMan probes used in study

| ***TaqMan Assay ID*** | **Transcript** |
| --- | --- |
| Hs99999901_s1 | 18S rRNA (normalization) |
| Hs00173317_m1 | Ankrd1 |
| Hs00220469_m1 | Ankrd2 |
| Hs00261590_m1 | Trim63 |
| Hs00979327_m1 | Trim55 |
| Hs00245918_m1 | Nbr1 |
| Hs00179935_m1 | FHL2 |
| Hs00185787_m1 | MLP |
| Hs00985784_g1 | TCAP |
| Hs00608023_m1 | Bcl-2 |
| **Hs00180269_m1** | Bax |
